# New avenues for functional neuroimaging: ultra-high field MRI and OPM-MEG

**DOI:** 10.1093/psyrad/kkab014

**Published:** 2021-12-09

**Authors:** Lang Qin, Jia-Hong Gao

**Affiliations:** Center for MRI Research, Academy for Advanced Interdisciplinary Studies, Peking University, Beijing 100871, China; McGovern Institute for Brain Research, Peking University, Beijing 100871, China; Center for MRI Research, Academy for Advanced Interdisciplinary Studies, Peking University, Beijing 100871, China; McGovern Institute for Brain Research, Peking University, Beijing 100871, China; Beijing City Key Laboratory for Medical Physics and Engineering, School of Physics, Peking University, Beijing 100871, China; National Biomedical Imaging Center, Peking University, Beijing 100871, China

**Keywords:** ultra-high field, MRI, OPM-MEG, neuroimaging

## Abstract

Functional brain imaging technology has developed rapidly in recent years. On the one hand, high-field 7-Tesla magnetic resonance imaging (MRI) has excelled the limited spatial resolution of 3-Tesla MRI, allowing us to enter a new world of mesoscopic imaging from the macroscopic imaging of human brain functions. On the other hand, novel optical pumping magnetometer-magnetoencephalography (OPM-MEG) has broken down the technical barriers of traditional superconducting MEG, which brings imaging of neuronal electromagnetic signals from cortical imaging to whole-brain imaging. This article aims to present a brief introduction regarding the development of conventional MRI and MEG technology, and, more importantly, to delineate that high-field MRI and OPM-MEG complement each other and together will lead us into a new era of functional brain imaging.

## Introduction

Human knowledge and the perception of the world originate from our brain. The human cerebral cortex can be, generally speaking, investigated and configured at three spatial scales, spanning from single neurons of several micrometres (microscopic scale), cortical laminae on a submillimetre level (mesoscopic scale), to cortical parcellation and white matter tracts on a centimetre level (macroscopic scale) (Dumoulin *et al*., 2018; Viessmann and Polimeni, 2021). Plausibly, investigating how whole-brain activities occur at all spatial scales is necessary to understanding human cognition and brain disorders. Since the late 1970s, magnetic resonance imaging (MRI) has progressed from initial grainy images to provide exquisite images of brain anatomy, function, and metabolites. Currently, irreplaceable advantages have made MRI integral to most neurologic evaluations. However, although MRI is a powerful non-invasive brain imaging technology, most MRI scanners can only reach the macroscopic scale. During the past 10 years, 3.0-Tesla (3-T) MRI scanners have been widely promoted and commonly used for many routine clinical applications, providing millimetre-level spatial resolution.

At the core, the most important communication vehicle between neurons in the human brain is electric current. Various modalities are used to obtain neural electrical information. Neuroelectrophysiology research and clinical brain surgery record electrical signals by directly implanting electrodes into the brain tissue, but such detection methods are invasive and cannot offer whole-brain coverage. The electroencephalogram (EEG), also known as scalp EEG, characterizes brain activity by recording the changes and distribution of electric potential on the scalp. Hans Berger reported the first case of spontaneous firing of neurons on the scalp of the human brain in 1929 (Berger, 1929). As the most common non-invasive brain neuroelectric signal detection method, EEG has been widely used in the clinical assessment of sleep, epilepsy, and some other neurological diseases. However, the electrical conductivity of the skull is much lower than that of other surrounding tissues, and thus greatly reduces the signal strength of extracranial EEG detection. Additionally, the dramatic change in electrical conductivity also seriously undermines and distorts the distribution of scalp potential. Therefore, despite the low cost and simple implementation, EEG is considerably limited by its signal distortion and poor spatial resolution, so it is mostly used for basic neuroscience research and auxiliary clinical assessment that must work with other diagnostic approaches.

In life sciences, electricity and magnetism as a set of deeply coupled signals carry a large amount of information about the activities of living organisms, and they are complementary to a certain extent. The propagation of nerve currents in the brain will generate a magnetic field, and the magnetic permeability values of different brain tissues and the skull are almost the same, indicating that the brain is basically ‘transparent’ to the propagation of magnetic fields (Okada *et al*., 1999). Such transparency provides a powerful driving force for the detection of the magnetic field of a brain nerve, to obtain a near-real-time nerve activity signal of the brain with minimal signal loss. However, such an ideal neuromagnetic signal detection comes at a price. The intensity of a typical brain magnetic field outside the scalp is between 10–100 fT (1 fT = 10^–15^ T), which is about one billionth of the Earth's magnetic field. How to realize the detection of extremely weak brain magnetic signals under the relatively huge background of the Earth's magnetic field and the dynamic interference of violently fluctuating external electromagnetic waves poses great challenges in terms of physical principles and technology. Around the same time that MRI emerged, David Cohen successfully detected magnetic brain signals generated by neuronal current in the human brain for the first time in history. He captured the alpha wave through multiple averaging and multi-turn induction coils in a specially constructed magnetic shielding room (Cohen, 1968). In 1972, Cohen further improved his method, using the Josephson junction superconducting quantum interferometer (SQUID) technology that has superior magnetic detection sensitivity for successfully detecting brain magnetic signals (Cohen, 1972). The SQUID system hallmarks the beginning of modern magnetoencephalography (MEG).

Here, this perspective article aims to briefly introduce conventional MRI and MEG technology, and, more importantly, to clarify an emerging trend of combining high-field MRI and the new generation of MEG.

### MRI development towards high-field

From the transition towards ultra-high field (e.g. 7 T and above), human brain MRI has profited tremendously. The signal-to-noise ratio (SNR) and contrast are two key factors determining MRI quality, and these two determinants increase with field strength. Higher SNR not only enables collection strategies for better image quality, but also enhances magnetic resonance contrasts based on magnetic susceptibility (i.e. blood-oxygenation-level dependent contrast, BOLD) (Balchandani and Naidich, 2015). In concrete terms, the BOLD contrast is established on the inhomogeneities of a microscopic magnetic field within or around blood vessels containing deoxyhaemoglobins, and such an effect will increase super-linearly with field strength. Importantly, evidence has proven that, while increased SNR and contrast also bring up technical challenges such as increased main field (B_0_) inhomogeneity, the benefits that endow MRI with the capacity to acquire images at higher spatial resolutions and/or reduced measurement time have greatly outweighed certain inevitable technical issues (Barth and Poser, 2011). In short, ultra-high field MRI can unprecedentedly offer non-invasive visualization of the brain with unparalleled detail, providing delicate anatomic and vascular information (Barisano *et al*., 2019; Dumoulin *et al*., 2018; Huber *et al*., 2017).

**Figure 1:**
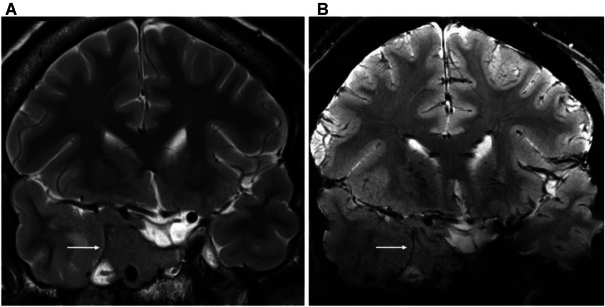
These images demonstrate how 7-T MRI provides more anatomical details of human brain than 3-T MRI. The white arrow denotes a homogenous sella macroadenoma. Comparison of 3 T (**A**) and 7 T (**B**) coronal T2-weighted images demonstrates far more detail of microvessels within the mass indicative of neovascularity, which is a known pathological feature of some macroadenomas (these images are adapted from Obusez *et al*., 2018).

During recent years, ultra-high field MRI for medical applications has attracted great interest. MRI scanners with field strengths of 7 T (up to 11.7 T) potentially allow for better detection and characterization of brain lesions and disorders, which thus can improve treatment solutions and help clarify the mechanisms underpinning different diseases (Balchandani and Naidich, 2015). In contrast with previous high-field clinical scanners, higher SNR and better image quality provided by ultra-high magnetic fields makes it possible to visualize small anatomical details and subtle pathological changes. Specifically, ultra-high-field MRI facilitates imaging of non-proton nuclei such as sodium, and boosts more precise detection of metabolites in spectroscopic MRI with improved spectral separation. All these advantages have a significant impact on a number of neurologic disorders and psychiatric conditions that are assumed to be related to subtle anatomical, functional, and metabolic abnormalizes, including epilepsy, multiple sclerosis, cerebrovascular disease, brain tumours, neurodegenerative diseases, and schizophrenia, etc. (Trattnig *et al*., 2018). Demonstrated in Fig. 1 is a comparison between 3- and 7-T MRI scans from a patient with pituitary macroadenoma (Obusez *et al*., 2018). Briefly, using these advanced imaging approaches, we will be able to capture such abnormal changes that are too subtle to be detected with conventional MRI scanners.

**Figure 2:**
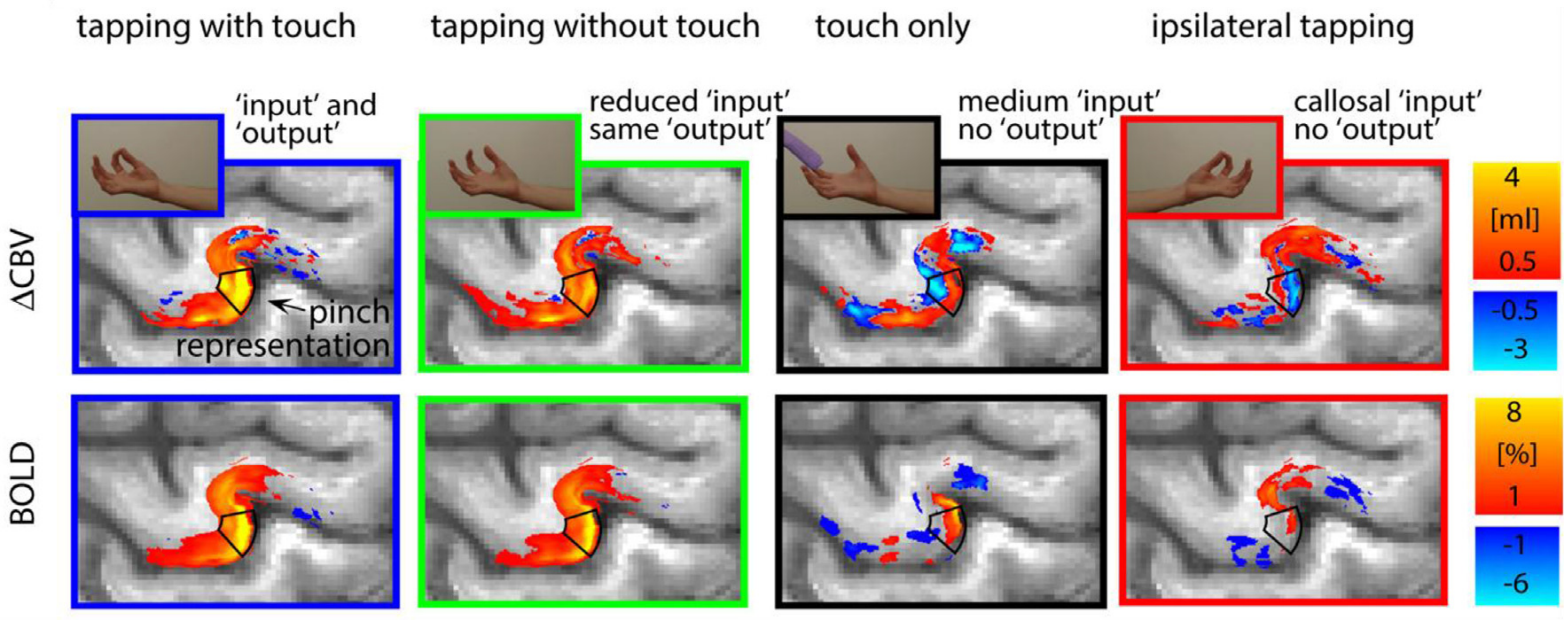
A recent laminar fMRI study calculated averaged group-level laminar fMRI responses in the motor cortex in response to four different sensorimotor tasks. As displayed in this figure, the four tasks evoked fMRI signals that varied with cortical depth in the thumb-index finger pinch motor area (these images are adapted from Huber *et al*., 2017).

Indeed, a vast body of research has demonstrated ultra-high-field MRI to be a powerful tool not only for advanced diagnostics and medical treatment, but also in the realms of general and cognitive neuroscience (Dumoulin, 2017). Among neuroimaging applications, functional MRI (fMRI) obtained with ultra-high-field scanners, in theory, will profit in more ways than just by an increase in SNR. As mentioned before, compared to lower field strengths, BOLD fMRI operating at ultra-high fields can afford a higher spatial resolution and better sensitivity, as well as superior specificity. Over the past 30 years, fMRI has become an irreplaceable approach that has advanced our understanding of human brain function. In particular, ultra-high field fMRI has enabled unparalleled visualization with near intrinsic resolution of functional detail at a laminar or columnar level in confined parts of the brain, boosting studies of fine-scale functional architecture such as cortical columns, layers, and subcortical nuclei (Dumoulin *et al*., 2018). Thus, fMRI at ultra-high magnetic fields is emerging as an unprecedented tool for exploring the fundamental processes performed in cortical micro-circuits and their corresponding interactions (e.g. feedforward and feedback processes) (Lawrence *et al*., 2019; Sharoh *et al*., 2019). Notably, Huber and his colleagues developed a multi-echo, BOLD-corrected vascular space occupancy (VASO) fMRI technique, which has been successfully used to investigate neurovascular responses during stimuli that elicit positive and negative BOLD responses human brain at 7 T (Huber *et al*., 2014; Huber *et al*., 2020); so far, researchers have uncovered laminar patterns involved in several high-level cognitive functions such as visual, working memory, and language processes (Finn *et al*., 2019; Huber *et al*., 2017; Sharoh *et al*., 2019). A laminar functional brain imaging study is illustrated in Fig. 2. Also, while group-level neuroimaging research is rooted in the motivation to increase sensitivity, meaningful differences in structure or functional organization can smear out subtle features as being averaged across individuals (Viessmann and Polimeni, 2021). Therefore, the increased statistical power of BOLD-fMRI at ultra-high fields may also contribute to finding individualized brain activity patterns by increasing statistical powers.

### Conventional MEG based on SQUID

Although it offers functional information with mesoscopic spatial resolution, in general, fMRI has two major limititations: (i) the temporal resolution is inadequate and (ii) fMRI only measures the haemodynamic response, so is unable to directly detect electrophysiological signals. In contrast, MEG can offset these limitations via direct capture of neuronal electrical activity with fine-grained temporal resolution at a millisecond level. However, spatial resolution of fMRI is higher than that of MEG source imaging. Thus, these two modalities are complementary to each other and the combination of information obtained from these two imaging technologies will provide a full picture of human brain function.

Commercialized MEG appeared in the 1980s, and developed from a single channel to a mature system with 200–300 channels covering the entire brain scale. Under laboratory conditions, the limit sensitivity of SQUID is about 1 ${\mathrm{fT}}/\sqrt {Hz} $, and the current commercial practical MEG SQUID detectors usually have a sensitivity of 2–3 ${\mathrm{fT}}/\sqrt {Hz} $. To maintain the superconducting working state of the system components, the magnetoencephalogram requires a Dewar device that contains liquid helium to maintain the ultra-low temperature and thermal insulation environment inside the system. A MEG system covering the whole brain consumes 10–20 l of liquid helium per day, and the MEG Dewar cavity can generally accommodate 70–90 l of liquid helium. Consequently, about one to two replenishments of liquid helium per week is necessary to assure the normal operation of the system. A replenishment process will take about 10 l of liquid helium, and the SQUID detection system is also in liquid helium. After the replenishment is completed, it takes several hours to reach a stable and usable state. At the same time, to maintain the ultra-low temperature inside the system under room temperature, the outermost layer of the Dewar device is evacuated to achieve the ideal thermal insulation effect. Generally, the thickness of the Dewar insulating vacuum layer is about 3 cm, which means there is a distance between the superconducting detector and the scalp where the magnetic field signal attenuates. In addition, the rigid Dewar helmet constrains the spatial arrangement of the detectors. Most MEG systems can only use a helmet array to match all participants. The mismatch between the helmet and the individual's head shape further enlarge the distance between the detector and the scalp, which has a particularly serious impact on studies with children.

High-sensitivity magnetic detectors need to work in a relatively low magnetic background environment, and the magnetic shielding system is also an indispensable part of the magnetoencephalogram. In view of the huge difference between the external magnetic field and the magnitude of the brain magnetic signal, the most direct way to reduce the interference is to place the brain magnetic detection equipment and the tested person in a closed magnetic shielded room (MSR) for data collection. The MSR of a commercial MEG is mostly composed of two or three layers of high-permeability alloy and high-conductivity alloy spliced ​​together. The side length is about 2–4 m, and the aluminium alloy is used as the framework. In addition to the passively working magnetically shielded outdoor room, the magnetoencephalogram also uses some other means to suppress noise, such as the active compensation coil (relying on the generation of a magnetic field that cancels out the external interference) in combination with the MSR. Although the superconducting MEG has become a commercial product, the expensive construction and maintenance costs of the equipment, the large signal attenuation distance, and the huge system space volume have severely restricted its popularization. At present, there are ~200 MEG systems installed worldwide, and the cumulative installed capacity in China is <12. The physics community has been looking for a new type of brain magnetic detection technology that does not rely on cryogenic superconducting refrigeration, has higher sensitivity, and miniaturization and integration that are easy to promote and use.

### Recent MEG development using OPM

**Figure 3:**
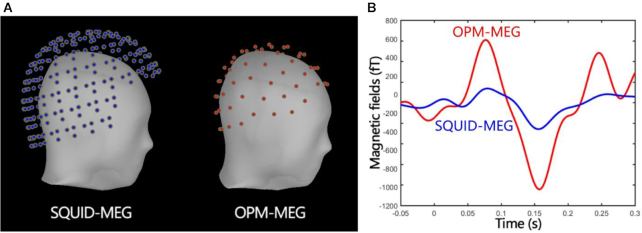
(**A**) The location of SQUID-MEG (left) and OPM-MEG (right) sensor array with respect to the scalp. (**B**) A comparison of signal amplitude of SQUID-MEG (blue) and OPM-MEG (red) under the same stimulus condition.

The atomic magnetometer [also known as an optical pumping magnetometer (OPM), atomic magnetometer, or atomic magnetometer] is a technology that uses the interaction of light and atoms to detect ultra-low magnetic fields, which is completely different from SQUID. Different from superconducting technology, the atomic magnetometer can work at room temperature and does not require refrigerants such as liquid helium. It is the best choice to replace traditional superconducting devices for brain magnetic detection. At present, the non-spin exchange relaxation atomic magnetometer device in the laboratory can achieve a sensitivity of 0.16 ${\mathrm{fT}}/\sqrt {Hz} $ (Dang *et al*., 2010), which is the most sensitive magnetic detection physical technology currently mastered by mankind, and its theoretical calculation sensitivity can reach 0.01 ${\mathrm{fT}}/\sqrt {Hz} $ or even lower (Kominis *et al*., 2003). More importantly, atomic magnetometers have the conditions for miniaturization and integration. The current miniaturized atomic magnetometers can be reduced to 1–2 cm in cross section, and their sensitivity can reach 10–20 ${\mathrm{fT}}/\sqrt {Hz} $ (Osborne *et al*., 2018). With the optimization of technology and control methods, this is constantly approaching the limit level of the laboratory. Since the atomic magnetometer does not need liquid helium to work, the new MEG device based on this technology will no longer be restricted by the huge Dewar device and become flexible, efficient, and cost-reduced, effectively solving the current superconducting brain problems with the magnetograph. Figure 3a shows the positional relationship between the superconducting magnetoencephalogram detector array and the atomic magnetometer magnetoencephalogram detection array reconstructed from the experiment and the scalp. After the distance limit of the vacuum insulation layer is no longer limited, the atomic magnetometer is almost close to the scalp and adapts to the contours of the individual's head, which directly leads to a substantial increase in the absolute intensity of the brain magnetic signal obtained by the detector. This advantage has been confirmed in multiple experiments (Fig. 3b).

The flexibility of the atomic magnetometer makes wearable MEG a possible direction. The University of Nottingham research group took the lead in realizing the recording of magnetoencephalogram signals within a certain range of motion in a MSR with an active compensation coil, and this was extended to people of different ages (Boto *et al*., 2017; Sheng *et al*., 2017). In addition, the new type of magnetic brain detector has opened up different solutions to magnetic shielding. Researchers have developed a high-performance compact cylindrical magnetic shielding system to fulfil the low-field requirement of the OPM when it is used to detect human brain magnetic fields induced by neuronal currents (He *et al*., 2019; Xia *et al*., 2006). New technologies for spatial registration of MEG and MRI based on optical scanning have been developed (Gu *et al*., 2021; Hill *et al*., 2019), which will help to further improve the clinical acceptance and promotion of MEG. The current atomic magnetometer technology, which combines miniaturization and high sensitivity, is in a period of rapid development. Admittedly, reasonable solutions are awaited to resolve and balance issues such as multi-channel signal crosstalk, response bandwidth, and gradiometer, etc. Once these problems related to brain magnetic signal acquisition and imaging are addressed, it is expected that a mature new type of MEG based on the atomic magnetometer (i.e. OPM-MEG) will be ready for users.

As a functional brain imaging modality with both high temporal resolution and high spatial resolution, MEG is currently an ideal technology that can obtain real-time neural activity at the whole-brain scale without trauma. In the development of systems based on superconducting magnetic detection technology for nearly 50 years, MEG has become an indispensable research tool for analysing brain functions. Clinical diagnosis of neurological diseases such as epilepsy and autism has shown its unique application value (Plummer *et al*., 2019; Roberts *et al*., 2010). The current new type of magnetic detection technology represented by the atomic magnetometer is expected to overcome the shortcomings of superconducting technology, leading to OPM-MEG that can obtain clearer brain activity signals and boasts more flexible detection and more diverse utility modes (Hill *et al*., 2020). Obviously, OPM-MEG will significantly expand the MEG application and lower the threshold of equipment installation. Besides and more importantly, since OPM-MEG is more flexible and more sensitive, it advances MEG from imaging the cortical activity to imaging both cortical and subcortical neuronal activity. It is universally acknowledged that SQUID MEG has very debatable and limited capacity for capturing deep sources, such as hippocampus and amygdala that play a critical role in memory and emotion (Bénar *et al*., 2021). In contrast, through creatively using OPM-MEG, researchers have obtained surprisingly improved signals of deep sources (Boto *et al*., 2021; Tierney *et al*., 2021).

## Conclusion

**Figure 4:**
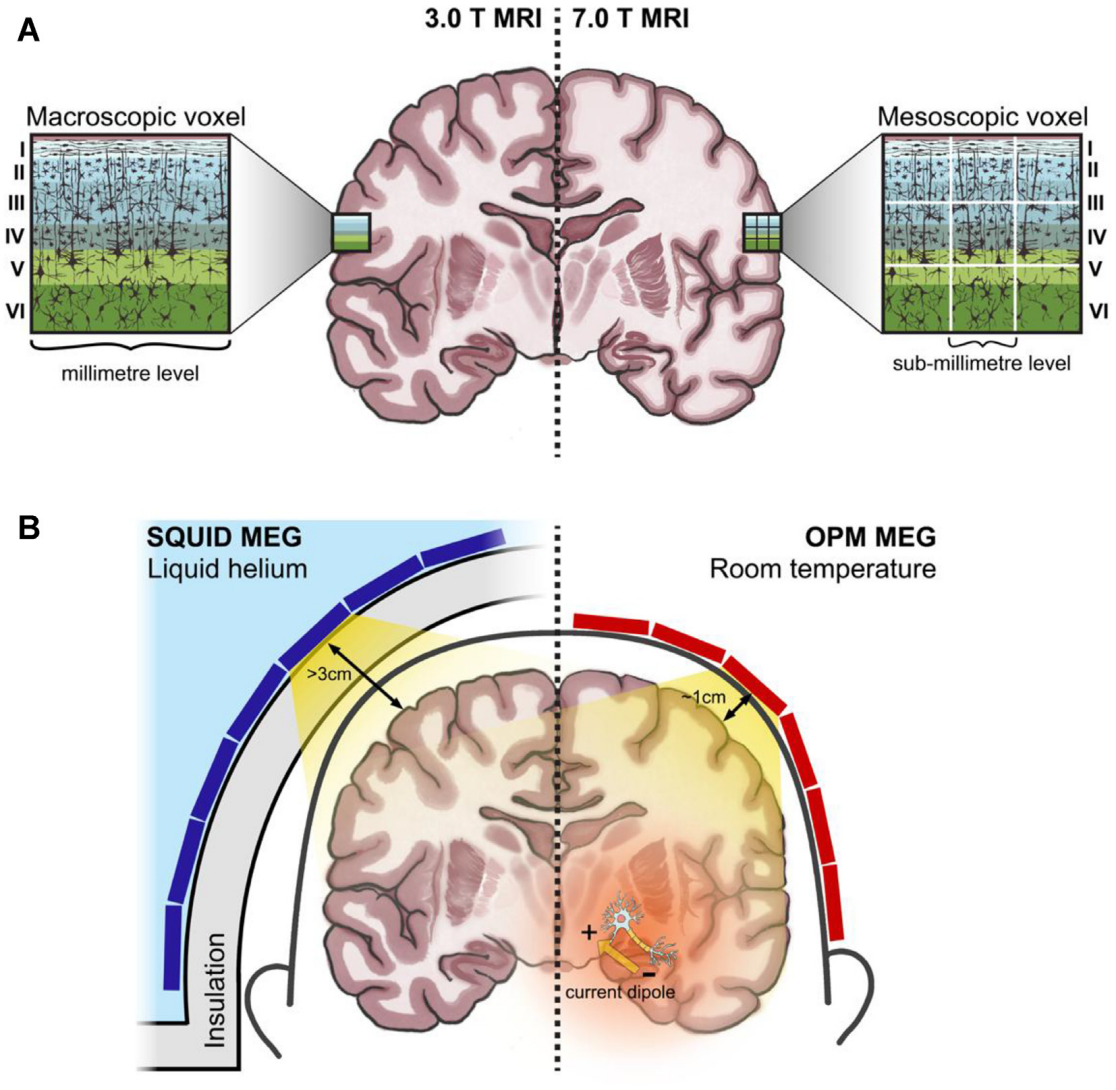
(**A**) Schematic demonstration of the comparison between the spatial resolution of 3- and 7-T MRI. For 3-T MRI, each voxel is of millimetre-level spatial resolution whereas 7-T MRI can reach submillimetre-level. Therefore, 3-T MRI cannot detect activities generated from different laminae of cerebral cortex but ultra-high-field MRI, such as 7-T MRI, has the potential to explore laminar phenomena. Six different colours denote, respectively, six cortical layers. (**B**) Schematic demonstration of the comparison between SQUID-MEG and OPM-MEG. Green blocks (left) and brown blocks (right) represent SQUID and OPM MEG sensors, respectively. OPM-MEG sensors have much better sensitivity to whole-brain neuronal signals.

In conclusion, high-field MRI and OPM-MEG complement each other and together will lead us into a new era of brain imaging. On the one hand, ultra-high field MRI owns unprecedented submillimetre spatial resolution, which will advance systems neuroscience from macroscopic towards mesoscopic brain functions (Fig. 4a); but MRI has limited temporal resolution and it merely indirectly reflects neuronal activity by assessing the hemodynamics. On the other hand, OPM-MEG brings MEG systems from superficial cortical imaging to whole-brain functional imaging (Fig. 4b), and it can complement these limitations of ultra-high field MRI via directly measuring electrophysiological signals with an optimal combination of terrific temporal resolution (millisecond-level) and spatial resolution. Notably, using these two methods will potentially help the neuroscience community reveal more mysteries of human brain.

## Supplementary Material

kkab014_Supplemental_Files
