# Supplementary material for: New avenues for functional neuroimaging: ultra-high field MRI and OPM-MEG
Source: Psychoradiology. 2021 Dec 9;1(4):165–71. doi: 10.1093/psyrad/kkab014 (PMC11025555; doi:10.1093/psyrad/kkab014)
Supplement: kkab014_Supplemental_Files [file kkab014_Supplemental_Files.zip › New_avenues_for_functional_neuroimaging_Gao_revised.docx]

**New avenues for functional neuroimaging:**

**Ultra-high field MRI and OPM-MEG**

**Lang Qin^1, 2^ and Jia-Hong Gao^1, 2, 3†^**

1. Center for MRI Research, Academy for Advanced Interdisciplinary Studies,
   Peking University, Beijing 100871, China
2. McGovern Institute for Brain Research, Peking University, Beijing 100871, China
3. Beijing City Key Lab for Medical Physics and Engineering, School of Physics, Peking University, Beijing 100871, China
4. National Biomedical Imaging Center, PekingUniversity, Beijing 100871, China

^†^Correspondence author (jgao@pku.edu.cn)

**Abstract:**

Functional brain imaging technology has developed rapidly in recent years. On the one hand, high-field 7T MRI has excelled the limited spatial resolution of 3T MRI, allowing us to enter a new world of mesoscopic imaging from the macroscopic imaging of human brain functions. On the other hand, the novel optical pumping magnetometer magnetoencephalography (OPM-MEG) has broken down the technical barriers of traditional superconducting MEG, which brings imaging of neuronal electromagnetic signals from cortical imaging to whole brain imaging. This article is aimed to present a brief introduction regarding the development of conventional MRI and MEG technology, and more importantly, to delineate that high-field MRI and OPM-MEG complement each other and together will lead us into a new era of functional brain imaging.

**Introduction**

Human knowledge and perception of the world are originated from our brain. Human cerebral cortex can be, generally speaking, investigated and configured at three spatial scales, spanning from single neurons with several micrometres (microscopic scale), cortical laminae on sub-millimetre level (mesoscopic scale) to cortical parcellation and white matter tracts on centimetre level (macroscopic scale) (Dumoulin et al. 2018; Viessmann and Polimeni 2021). Plausibly, investigating how whole-brain activities that occur at all spatial scales is necessary to understanding human cognition and brain disorders. Since the late 1970s, magnetic resonance imaging (MRI) has progressed from initial grainy images to provide exquisite images of brain anatomy, function and metabolites. Currently, irreplaceable advantages have made MRI integral to mostly all neurologic evaluations. However, MRI as a powerful non-invasive brain imaging technology, most MRI scanners can only reach macroscopic scale. During past 10 years, 3.0 Tesla (3T) MRI scanners have been widely promoted and commonly used for many routine clinical applications, providing millimetre-level spatial resolution.

At the core, the most important communication vehicle between neurons in the human brain is electric current. Various modalities are used to obtain neural electrical information. Neuroelectrophysiology research and clinical brain surgery record electrical signals by directly implanting electrodes into the brain tissue, but such detection methods are invasive and cannot offer whole-brain coverage. Electroencephalogram (EEG), known as scalp electroencephalogram, characterizes brain activity by recording the changes and distribution of electric potential on the scalp. Hans Berger reported the first case of spontaneous firing of neurons on the scalp of the human brain in 1929 (Berger 1929). As the most common non-invasive brain neuroelectric signal detection method, EEG has been widely used in the clinical assessment of sleep, epilepsy and some other neurological diseases. However, the electrical conductivity of the skull is much lower than that of other surrounding tissues and thus greatly reduces the signal strength of extracranial EEG detection. Additionally, the dramatic change in electrical conductivity also seriously undermines and distorts the distribution of scalp potential. Therefore, despite the low cost and simple implementation, EEG is considerably limited by its signal distortion and poor spatial resolution, mostly utilized for basic neuroscience research and auxiliary clinical assessment that must work with other diagnostic approaches.

In life sciences, electricity and magnetism, as a set of deeply coupled signals, carry a large amount of information about the activities of living organisms, and they are complementary to a certain extent. The propagation of nerve currents in the brain will generate a magnetic field, and the magnetic permeability values of different brain tissues and the skull are almost the same, indicating that the brain is basically ‘transparent’ to the propagation of magnetic fields (Okada et al. 1999). Such transparency provides a powerful driving force for the detection of the magnetic field of the brain nerve: to obtain a near-real-time nerve activity signal of the brain with minimal signal loss. However, such an ideal neuromagnetic signal detection comes at a price. The intensity of a typical brain magnetic field outside the scalp is between 10-100 fT (1 fT = 10^-15^ Tesla), which is about one billionth of the earth's magnetic field. How to realize the detection of extremely weak brain magnetic signals under the relatively huge background of the earth's magnetic field and the dynamic interference of violently fluctuating external electromagnetic waves poses great challenges in terms of physical principles and technology. Around the same time when MRI emerged, David Cohen successfully detected magnetic brain signals generated by neuronal current in human brain for the first time in history. He captured the alpha wave through multiple averaging and multi-turn induction coils in a specially constructed magnetic shielding room (Cohen 1968). In 1972, Cohen further improved his method, using the Josephson junction superconducting quantum interferometer (SQUID) technology that owns superior magnetic detection sensitivity successfully to detect brain magnetic signals (Cohen 1972). The SQUID system hallmarks the beginning of modern magnetoencephalography (MEG).

Here, this perspective article is aimed to briefly introduce conventional MRI and MEG technology, and more importantly, to clarify an emerging trend of the combining high-field MRI and new generation of MEG.

**MRI development towards high-field**

From the transition towards ultra-high field (e.g., 7T and above), human brain MRI has profited tremendously. The signal-to-noise ratio (SNR) and contrast are two key factors determining MR image quality, and these two determinants increase with field strength. Higher SNR non only enable collection strategies for better image quality, but also enhances MR contrasts based on magnetic susceptibility (i.e., blood-oxygenation-level dependent contrast, BOLD) (Balchandani and Naidich 2015). In concrete terms, the BOLD contrast is established upon the inhomogeneities of microscopic magnetic field within or around blood vessels containing deoxyhaemoglobins, and such effect will increase super-linearly with field strength. Importantly, evidence has proven that, while increased SNR and contrast also bring up technical challenges such as the increased main field (B_0_) inhomogeneity, these benefits that endow MRI with the capacity to acquire images with higher spatial resolutions and/or reduced measurement time have greatly outweighed certain inevitable technical issues (Barth and Poser 2011). In short, ultra-high field MR imaging can unprecedentedly offer non-invasive visualisation of the brain with unparalleled detail, providing delicate anatomic and vascular information (Barisano et al. 2019; Dumoulin et al. 2018; Huber et al. 2017).

During recent years, ultra-high field MRI for medical application has attracted great interest. MRI scanners with field strengths of 7T (up to 11.7T) potentially allow for better detection and characterization of brain lesions and disorders, which thus can improve treatment solutions and help clarify the mechanisms underpinning different diseases (Balchandani and Naidich 2015). In contrast with previous high-field clinical scanners, higher SNR and better image quality provided by ultra-high magnetic fields makes it possible to visualise small anatomical detail and subtle pathological changes. Specifically, ultra-high-field MRI facilitates imaging of nonproton nuclei such as sodium and boosts more precise detection of metabolites in MR spectroscopic imaging with improved spectral separation. All these advantages have a significant impact on a number of neurologic disorders and psychiatric conditions that are assumed to be related to subtle anatomical, functional and metabolic abnormalizes, including epilepsy, multiple sclerosis, cerebrovascular disease, brain tumours, neurodegenerative diseases, and schizophrenia etc (Trattnig et al. 2018). As demonstrated in Figure 1 is a comparison between 3T and 7T MR images from a patient with pituitary macroadenoma (Obusez et al. 2018). Briefly, using these advanced imaging approaches, we will be able to capture such abnormal changes that are too subtle to be detected with conventional MRI scanners.

Indeed, a vast body of research has demonstrated ultra-high-field MRI a powerful tool not only for advanced diagnostics and medical treatment, but also in realms of general and cognitive neuroscience (Dumoulin 2017). Among the neuroimaging applications, functional MRI (fMRI) obtained with ultra-high-field scanners in theory will profit in more ways than just by an increase SNR. As mentioned before, compared to lower field strengths, BOLD fMRI operating at ultra-high fields can afford higher spatial resolution, better sensitivity as well as superior specificity. Over past 30 years, fMRI has become an irreplaceable approach that advanced our understanding of human brain function. Particularly, ultra-high field fMRI has enabled unparalleled visualization with near intrinsic resolution of functional detail at a laminar or columnar level in confined parts of the brain, boosting studies of fine-scale functional architecture such as cortical columns, layers, and subcortical nuclei (Dumoulin et al. 2018). Thus, fMRI at ultra-high magnetic fields is emerging as an unprecedented tool for exploring the fundamental processes performed in cortical micro-circuits and their corresponding interactions (e.g., feedforward and feedback processes) (Lawrence et al. 2019; Sharoh et al. 2019). Notably, Huber and his colleagues developed a multi-echo, BOLD-corrected vascular space occupancy (VASO) fMRI technique, which has been successfully used to investigate neurovascular responses during stimuli that elicit positive and negative BOLD responses human brain at 7T (Huber et al. 2014; Huber et al. 2020); by far, researchers have uncovered laminar patterns involved in several high-level cognitive functions such as visual, working memory and language processes (Finn et al. 2019; Huber et al. 2017; Sharoh et al. 2019). A laminar functional brain imaging study is illustrated in Figure 2. Also, while group-level neuroimaging research is rooted in the motivation to increase sensitivity, meaningful differences in structure or functional organization can smear out subtle features as being averaged across individuals (Viessmann and Polimeni 2021). Therefore, the increased statistical power of BOLD-fMRI at ultra-high fields may also contribute to finding individualized brain activity patterns by increasing statistical powers.

**Conventional MEG based on SQUID**

Although it offers functional information with mesoscopic spatial resolution, in general, fMRI has two major limititations: (1) the temporal resolution is inadequate and (2) fMRI only measures the hemodynamic response and is unable to directly detect electrophysiological signals. In contrast, MEG can offset these limitations via directly capture neuronal electrical activity with fine-grained temporal resolution at millisecond level. However, spatial resolution of fMRI is higher than that of MEG source imaging. Thus, these two modalities are complementary to each other and combination of informaton obtained from these two imaging technologies will provide a full picture of human brain function.

Commercialized MEG appeared in the 1980s, and developed from a single channel to a mature system with 200-300 channels covering the entire brain scale. Under laboratory conditions, the limit sensitivity of SQUID is about 1 $fT/\sqrt{Hz}$, and the current commercial practical magnetoencephalogram SQUID detectors usually have a sensitivity of 2-3 $fT/\sqrt{Hz}$. To maintain the superconducting working state of the system components, the magnetoencephalogram requires a Dewar device which contains the liquid helium to maintain the ultra-low temperature and thermal insulation environment inside the system. A MEG system covering the whole brain consumes 10-20 litres of liquid helium per day, and the MEG Dewar cavity can generally accommodate 70-90 litres of liquid helium. Consequently, about 1-2 replenishment of liquid helium per week is necessary to assure the normal operation of the system. A replenishment process will take about 10 litres of liquid helium, and the SQUID detection system is in liquid helium. After the replenishment is completed, it takes several hours to reach a stable and usable state. At the same time, to maintain the ultra-low temperature inside the system under room temperature, the outermost layer of the Dewar device is evacuated to achieve the ideal thermal insulation effect. Generally, the thickness of the Dewar insulating vacuum layer is about 3 cm, which means there is a distance between the superconducting detector and the scalp where the magnetic field signal attenuates. In addition, the rigid Dewar helmet constrains the spatial arrangement of the detectors. Most MEG systems can only use a helmet array to match all subjects. The mismatch between the helmet and the individual’s head shape further enlarge the distance between the detector and the scalp, which has a particularly serious impact on studies with children.

High-sensitivity magnetic detectors need to work in a relatively low magnetic background environment, and the magnetic shielding system is also an indispensable part of the MEG. In view of the huge difference between the external magnetic field and the magnitude of the brain magnetic signal, the most direct way to reduce the interference is to place the brain magnetic detection equipment and the tested person in a closed magnetic shielded room (MSR) for data collection. The MSR of a commercial magnetoencephalography is mostly composed of two or three layers of high-permeability alloy and high-conductivity alloy spliced ​​together. The side length is about 2-4 metres, and the aluminium alloy is used as the framework. In addition to the passively working magnetically shielded outdoor room, the magnetoencephalogram also uses some other means to suppress noise, such as the active compensation coil (relying on the generation of a magnetic field that cancels out the external interference) in combination with the magnetic shielded room. Although the superconducting MEG has become a commercial product, the expensive construction and maintenance costs of the equipment, the large signal attenuation distance, and the huge system space volume have severely restricted its popularization. At present, there are about two hundred MEG systems installed worldwide, and the cumulative installed capacity in China is less than 12. The physics community has been looking for a new type of brain magnetic detection technology that does not rely on cryogenic superconducting refrigeration and has higher sensitivity and miniaturization and integration that is easy to promote and utilize.

**Recent MEG development using OPM**

Atomic magnetometer (also known as optical pumping magnetometer, OPM, atomic magnetometer, atomic magnetometer) is a technology that uses the interaction of light and atoms to detect ultra-low magnetic fields, which is completely different from superconducting quantum interferometers. Different from superconducting technology, the atomic magnetometer can work at room temperature and does not require refrigerants such as liquid helium. It is the best choice to replace traditional superconducting devices for brain magnetic detection. At present, the non-spin exchange relaxation atomic magnetometer device in the laboratory can achieve a sensitivity of 0.16 $fT/\sqrt{Hz}$ (Dang et al. 2010), which is the most sensitive magnetic detection physical technology currently mastered by mankind, and its theoretical calculation sensitivity can reach 0.01 $fT/\sqrt{Hz}$ or even lower (Kominis et al. 2003). More importantly, atomic magnetometers have the conditions for miniaturization and integration. The current miniaturized atomic magnetometers can be reduced to 1-2 cm in cross section, and their sensitivity can reach 10-20 $fT/\sqrt{Hz}$ (Osborne et al. 2018). With the optimization of technology and control methods, it is constantly approaching the limit level of the laboratory. Since the atomic magnetometer does not need liquid helium to maintain its work, the new MEG device based on this technology will no longer be restricted by the huge Dewar device and become flexible, efficient, and cost-reduced, effectively solving the current superconducting brain problems with the magnetograph. Figure 3a shows the positional relationship between the superconducting magnetoencephalogram detector array and the atomic magnetometer magnetoencephalogram detection array reconstructed from the experiment and the scalp. After the distance limit of the vacuum insulation layer is no longer limited, the atomic magnetometer can almost close to the scalp and adapt to the contours of the individual's head, which directly leads to a substantial increase in the absolute intensity of the brain magnetic signal obtained by the detector. This advantage has been confirmed in multiple experiments (Figure 3b).

The flexibility of the atomic magnetometer makes wearable MEG a possible direction. The University of Nottingham research group took the lead in realizing the recording of magnetoencephalogram signals within a certain range of motion in a magnetic shielded room with an active compensation coil, and it was extended to people of different ages (Boto et al. 2017; Sheng et al. 2017). In addition, the new type of magnetic brain detector has opened up different solutions to magnetic shielding. Researchers have developed a high-performance compact cylindrical magnetic shielding system to fulfil the low-field requirement of the OPM when it is used to detect human brain magnetic fields induced by neuronal currents (He et al. 2019; Xia et al. 2006). New technologies for spatial registration of MEG and magnetic resonance imaging based on optical scanning have been developed (Gu et al. 2021; Hill et al. 2019), which will help to further improve the clinical acceptance and promotion of MEG. The current atomic magnetometer technology, which combines miniaturization and high sensitivity, is in a period of rapid development. Admittedly, reasonable solutions are awaited to be given to resolve and balance issues such as multi-channel signal crosstalk, response bandwidth and gradiometer etc. Once these problems related to brain magnetic signal acquisition and imaging are addressed, it is expected that a mature new type of MEG based on the atomic magnetometer (i.e., OPM-MEG) will be ready to users.

As a functional brain imaging modality with both high temporal resolution and high spatial resolution, MEG is currently an ideal technology that can obtain real-time neural activity at the whole brain scale without trauma. In the development of systems based on superconducting magnetic detection technology for nearly 50 years, MEG has become an indispensable research tool for analysing brain functions. Clinical diagnosis of neurological diseases such as epilepsy and autism has shown its unique application value (Plummer et al. 2019; Roberts et al. 2010). The current new type of magnetic detection technology represented by the atomic magnetometer is expected to overcome the shortcomings of superconducting technology, leading to OPM-MEG that can obtain clearer brain activity signals and boasts more flexible detection and more diverse utility modes (Hill et al. 2020). Obviously, OPM-MEG will significantly expand the MEG application and lower the threshold of equipment installation. Besides the fact and more importantly, since OPM-MEG is more flexible and more sensitive, it advances MEG from imaging the cortical activity to imaging both cortical and sub-cortical neuronal activity. It is universally acknowledged that SQUID MEG has very debatable and limited capacity of capturing deep sources, such as [hippocampus](https://www.sciencedirect.com/topics/neuroscience/hippocampus) and [amygdala](https://www.sciencedirect.com/topics/medicine-and-dentistry/amygdala) that play a critical role in memory and emotion (Bénar et al. 2021). In contrast, through creatively using OPM-MEG, researchers have obtained surprisingly improved signals of deep sources (Boto et al. 2021; Tierney et al. 2021).

**Conclusion**

In conclusion, high-field MRI and OPM-MEG complement each other and together will lead us into a new era of brain imaging. On the one hand, ultra-high field MRI owns unprecedented sub-millimetre spatial resolution, which will advance systems neuroscience from macroscopic towards mesoscopic brain functions (Figure 4a); but MRI has limited temporal resolution and it merely indirectly reflects neuronal activity by assessing the hemodynamics. On the other hand, OPM-MEG brings MEG systems from superficial cortical imaging to whole-brain functional imaging (Figure 4b), and it can complement these limitations of ultra-high field MRI via directly measuring electrophysiological signals with optimal combination of terrific temporal resulition (millisecond-level) and spatial resulotion. Notably,. Utilizing these two methods potentially will help the neuroscience community reveal more mysteries of human brain.

**References**

Balchandani, P and Naidich, TP (2015), 'Ultra-high-field MR neuroimaging', *American Journal of Neuroradiology,* 36 (7), 1204-15.

Barisano, Giuseppe, et al. (2019), 'Clinical 7 T MRI: Are we there yet? A review about magnetic resonance imaging at ultra-high field', *The British journal of radiology,* 92 (1094), 20180492.

Barth, Markus and Poser, Benedikt A (2011), 'Advances in high-field BOLD fMRI', *Materials,* 4 (11), 1941-55.

Bénar, C-G, et al. (2021), 'Detection and localization of deep sources in magnetoencephalography: A review', *Current Opinion in Biomedical Engineering*, 100285.

Berger, Hans (1929), 'Über das elektroenkephalogramm des menschen', *Archiv für psychiatrie und nervenkrankheiten,* 87 (1), 527-70.

Boto, Elena, et al. (2017), 'A new generation of magnetoencephalography: Room temperature measurements using optically-pumped magnetometers', *NeuroImage,* 149, 404-14.

Boto, Elena, et al. (2021), 'Measuring functional connectivity with wearable MEG', *NeuroImage,* 230, 117815.

Cohen, David (1968), 'Magnetoencephalography: evidence of magnetic fields produced by alpha-rhythm currents', *Science,* 161 (3843), 784-86.

--- (1972), 'Magnetoencephalography: detection of the brain's electrical activity with a superconducting magnetometer', *Science,* 175 (4022), 664-66.

Dang, HB, Maloof, Adam C, and Romalis, Michael V (2010), 'Ultrahigh sensitivity magnetic field and magnetization measurements with an atomic magnetometer', *Applied Physics Letters,* 97 (15), 151110.

Dumoulin, Serge O (2017), 'Layers of neuroscience', *Neuron,* 96 (6), 1205-06.

Dumoulin, Serge O, et al. (2018), 'Ultra-high field MRI: Advancing systems neuroscience towards mesoscopic human brain function', *Neuroimage,* 168, 345-57.

Finn, Emily S, et al. (2019), 'Layer-dependent activity in human prefrontal cortex during working memory', *Nature neuroscience,* 22 (10), 1687-95.

Gu, Wenyu, et al. (2021), 'Automatic coregistration of MRI and on-scalp MEG', *Journal of Neuroscience Methods,* 358, 109181.

He, Kaiyan, et al. (2019), 'A high-performance compact magnetic shield for optically pumped magnetometer-based magnetoencephalography', *Review of Scientific Instruments,* 90 (6), 064102.

Hill, Ryan M, et al. (2019), 'A tool for functional brain imaging with lifespan compliance', *Nature communications,* 10 (1), 1-11.

Hill, Ryan M, et al. (2020), 'Multi-channel whole-head OPM-MEG: Helmet design and a comparison with a conventional system', *NeuroImage,* 219, 116995.

Huber, Laurentius, et al. (2014), 'Investigation of the neurovascular coupling in positive and negative BOLD responses in human brain at 7 T', *Neuroimage,* 97, 349-62.

Huber, Laurentius, et al. (2017), 'High-resolution CBV-fMRI allows mapping of laminar activity and connectivity of cortical input and output in human M1', *Neuron,* 96 (6), 1253-63. e7.

Huber, Laurentius, et al. (2020), 'Layer-dependent functional connectivity methods', *Progress in Neurobiology*, 101835.

Kominis, IK, et al. (2003), 'A subfemtotesla multichannel atomic magnetometer', *Nature,* 422 (6932), 596-99.

Lawrence, Samuel JD, et al. (2019), 'Laminar fMRI: Applications for cognitive neuroscience', *Neuroimage,* 197, 785-91.

Obusez, Emmanuel C, et al. (2018), '7T MR of intracranial pathology: Preliminary observations and comparisons to 3T and 1.5 T', *Neuroimage,* 168, 459-76.

Okada, Yoshio C, Lahteenmäki, Airi, and Xu, Chibing (1999), 'Experimental analysis of distortion of magnetoencephalography signals by the skull', *Clinical neurophysiology,* 110 (2), 230-38.

Osborne, J, et al. (2018), 'Fully integrated standalone zero field optically pumped magnetometer for biomagnetism', *Steep Dispersion Engineering and Opto-Atomic Precision Metrology XI* (10548: International Society for Optics and Photonics), 105481G.

Plummer, Chris, et al. (2019), 'Interictal and ictal source localization for epilepsy surgery using high-density EEG with MEG: a prospective long-term study', *Brain,* 142 (4), 932-51.

Roberts, Timothy PL, et al. (2010), 'MEG detection of delayed auditory evoked responses in autism spectrum disorders: towards an imaging biomarker for autism', *Autism Research,* 3 (1), 8-18.

Sharoh, Daniel, et al. (2019), 'Laminar specific fMRI reveals directed interactions in distributed networks during language processing', *Proceedings of the National Academy of Sciences,* 116 (42), 21185-90.

Sheng, Jingwei, et al. (2017), 'Magnetoencephalography with a Cs-based high-sensitivity compact atomic magnetometer', *Review of Scientific Instruments,* 88 (9), 094304.

Tierney, Tim M, et al. (2021), 'Mouth magnetoencephalography: A unique perspective on the human hippocampus', *NeuroImage,* 225, 117443.

Trattnig, Siegfried, et al. (2018), 'Key clinical benefits of neuroimaging at 7 T', *Neuroimage,* 168, 477-89.

Viessmann, Olivia and Polimeni, Jonathan R (2021), 'High-resolution fMRI at 7 Tesla: challenges, promises and recent developments for individual-focused fMRI studies', *Current Opinion in Behavioral Sciences,* 40, 96-104.

Xia, H, et al. (2006), 'Magnetoencephalography with an atomic magnetometer', *Applied Physics Letters,* 89 (21), 211104.


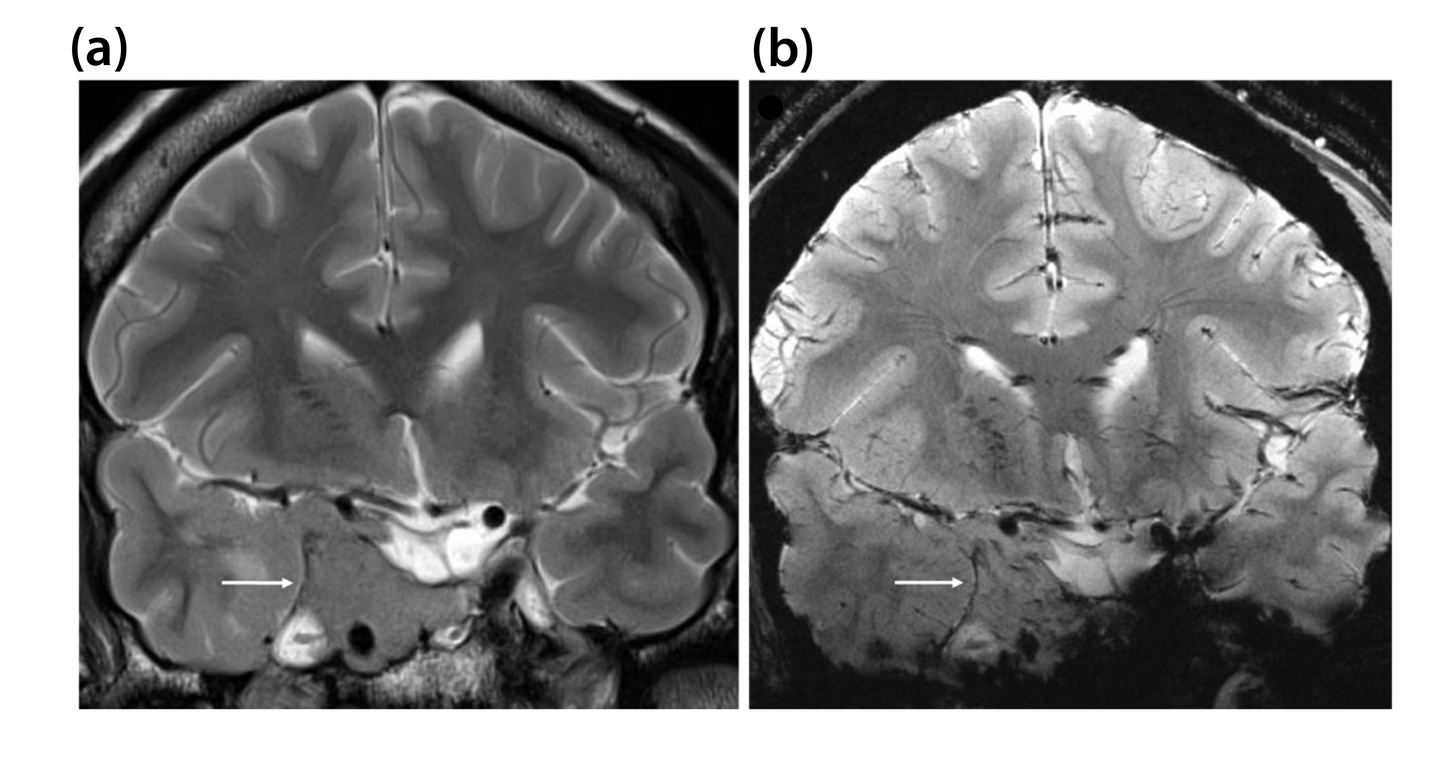


**Figure 1.** These images demonstrated how 7T MRI provides more anatomical details of human brain than 3T MRI. The white arrow denotes homogenous [sella](https://www.sciencedirect.com/topics/medicine-and-dentistry/sella-turcica) [macroadenoma](https://www.sciencedirect.com/topics/medicine-and-dentistry/macroadenoma). In comparison with 3T (**a**) and 7T (**b**) coronal T2-weighted images demonstrate much more detail of [microvessels](https://www.sciencedirect.com/topics/medicine-and-dentistry/microvessel) within the mass indicative of neovascularity, which is a known pathological feature of some macroadenomas (These images are adapted from Obusez et al. 2018).


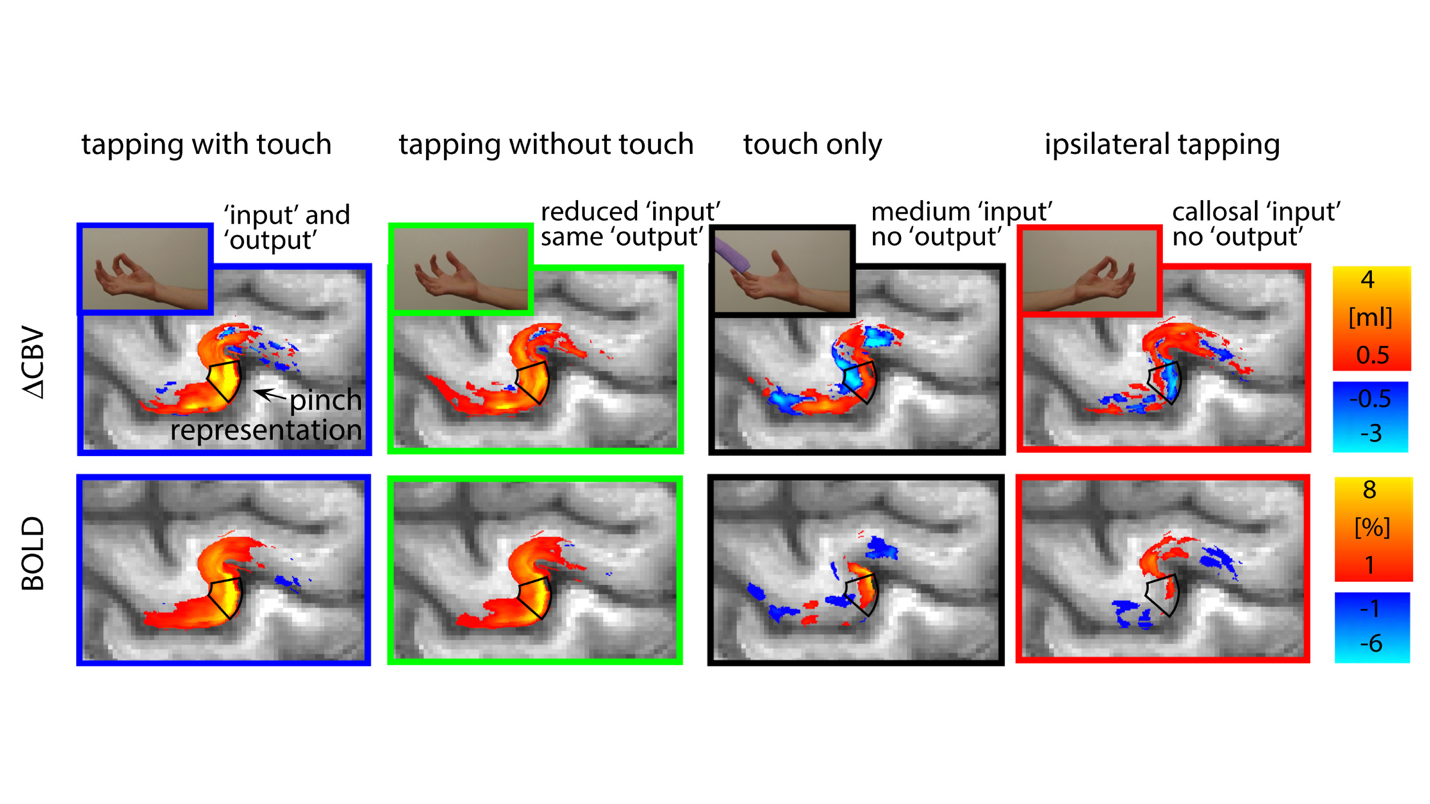


**Figure 2.** A recent laminar fMRI study calculated averaged group-level laminar fMRI responses in the motor cortex in response to four different sensorimotor tasks. As displayed in this figure, the four tasks evoked fMRI signals that varied with cortical depth in the thumb-index finger pinch motor area (These images are adapted from Huber et al 2017).


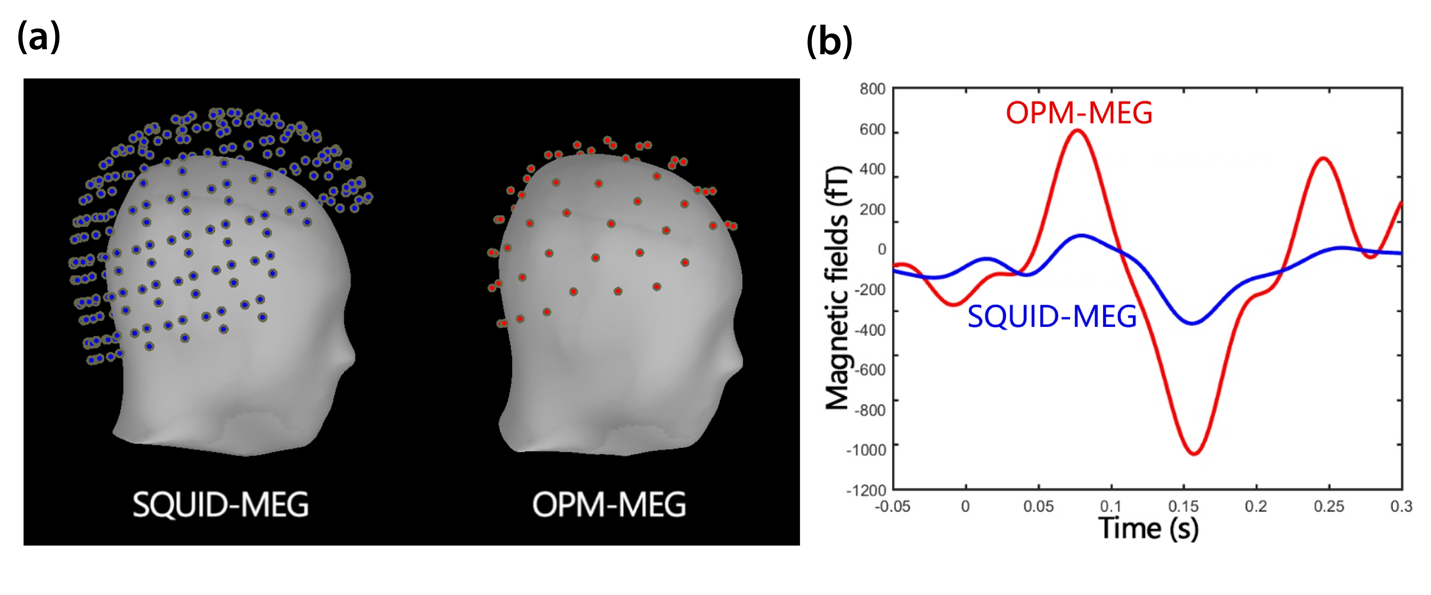


**Figure 3.** (**a**) The location of SQUID-MEG (left) and OPM-MEG (right) sensor array with respect to the scalp. (**b**) A comparison of signal amplitude of SQUID-MEG (blue) and OPM-MEG (red) under the same stimulus condition.


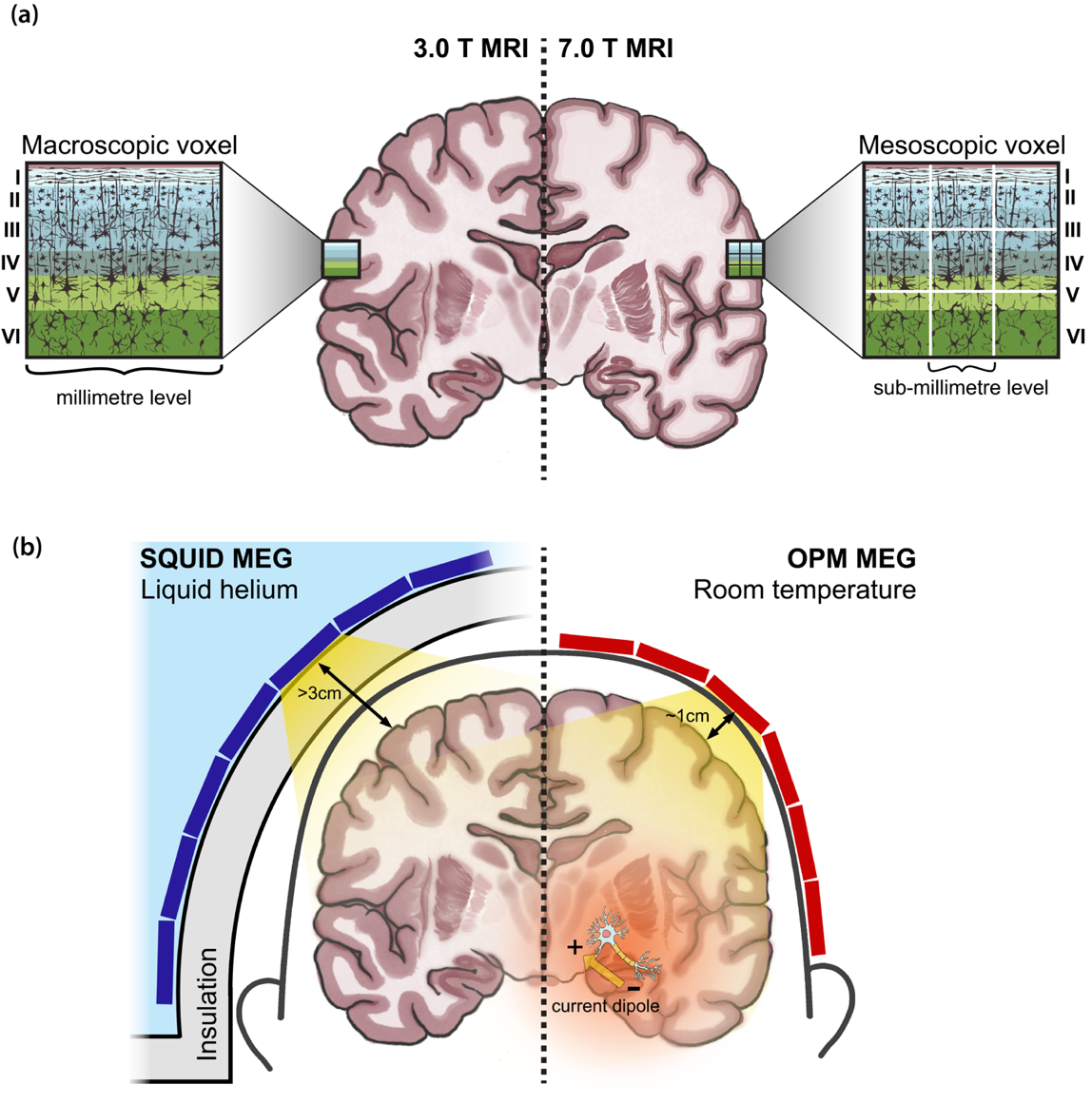


**Figure 4.** (**a**) Schematic demonstration of the comparison between the spatial resolution of 3T MRI and 7T MRI. For 3T MRI, each voxel is of millimetre-level spatial resolution whereas 7 T MRI can reach sub-millimetre-level. Therefore, 3T MRI can by no means detect activities generated from different laminae of cerebral cortex but ultra-high-field MRI such as 7T MRI owns the potential to explore laminar phenomena. Six different colours denote respectively six cortical layers. (**b**) Schematic demonstration of the comparison between SQUID-MEG and OPM-MEG. Green blocks (left) and brown blocks (right) represent SQUID and OPM MEG sensors, respectively. OPM-MEG sensors have much better sensitivity to whole-brain neuronal signals.
