# Supplementary material for: New avenues for functional neuroimaging: ultra-high field MRI and OPM-MEG
Source: Psychoradiology. 2021 Dec 9;1(4):165–71. doi: 10.1093/psyrad/kkab014 (PMC11025555; doi:10.1093/psyrad/kkab014)
Supplement: kkab014_Supplemental_Files [file kkab014_Supplemental_Files.zip › Response to the Editors and Reviewers.docx]

**Response to the Editors and Reviewers**

Reviewers' comments are in *italics,* and our responses are in **bold**. Additionally, all changes in the manuscript are written in blue.

**We appreciate the insightful and constructive comments from the two reviewers. We have carefully addressed all of the comments, and our detailed point-by-point responses are listed below.**

***Reviewer: 1***

*Comments to the Author
In this article, the authors reviewed recent advancements in ultra-high field MRI and OPM-MEG. The paper is well written and systematically describes their history, technical development, and applications. I only have two minor suggestions without any major concern.*

*1) The authors discussed the topics of high-field MRI and OPM-MEG, separately, without talking any link between these two techniques. If either of them is so powerful, why do we need to develop both high-field MRI and OPM-MEG. How can high-field MRI and OPM-MEG complement each other? For example,* *high-field MRI may provide higher spatial resolution while OPM-MEG can do better in providing high temporal resolution information.*

**We thank the reviewer for this suggestion. We have added explanatory description about the differences between MRI and MEG as the first paragraph of Conventional MEG based on SQUID and the Conclusion part (Pages 7, 12).**

*2) The author may consider adding section titles to better structure the article and help reader follow the content better, e.g.,* *MRI development towards high-field, recent MEG development using OPM, how high-field MRI and OPM-MEG complement each other, conclusion.*

**We thank the reviewer for this suggestion. We have added sub-titles and reframed the Introduction (Pages 2-4).**
 ***Reviewer: 2*** *Comments to the Author
This is a good review to learn the frontier of brain signal detection. However, there are still two points that need to be revised to better benefit readers*

*1, The format of the manuscript should be modified to meet the requirements of the magazine for publication, such as  an abstract is needed in this review, etc.*

**We thank the reviewer for this suggestion. We have checked the requirements of the journal and made modifications accordingly.**

2, The author also introduces the progress of high field MRI and MEG in brain imaging, if adding some content about the consistency or comparison between the two imaging tool it will make the article more valuable.

**We have a brief description about the consistency between these two imaging tools to the Conventional MEG based on SQUID and the Conclusion part (Pages 7, 12).**
